# THE RELATIONSHIP BETWEEN GLOBAL AND DAILY LEVELS OF PAIN AND RELIGIOUS COPING

**DOI:** 10.1093/geroni/igz038.1930

**Published:** 2019-11-08

**Authors:** Katherine L Cheesman, Brian Cox, Dylan M Smith, Patricia A Parmelee

**Affiliations:** 1 The University of Alabama, Tuscaloosa, Alabama, United States; 2 Alabama Research Institute of Aging, Tuscaloosa, Alabama, United States; 3 Stony Brook University, Stony Brook, New York, United States

## Abstract

Objective: This research examines associations between global and daily levels of pain and the use of religious coping strategies among African American (AA) and non-Hispanic White (NHW) older adults with physician-confirmed knee osteoarthritis (OA). Methods: As part of a larger study of racial/ethnic differences in everyday quality of life with OA, 125 persons over the age of 50 completed a global measure of religious coping using the Brief RCOPE. Daily variability in pain and coping was assessed using a daily diary methodology consisting of 4 daily phone calls over 7 days. Hypotheses: Demographic characteristics (sex, race) were expected to predict religious coping at both the global and daily levels. Specifically, we expected women and AAs would use more religious coping than their male and NHW counterparts. Further, daily levels of pain were predicted to correlate with daily levels of coping. Results: AAs were found to engage in significantly more religious coping behaviors than NHWs at the global, but not daily, level. An intraclass correlation coefficient of .316 indicated sufficient within-person variability for the use of multi-level modeling to determine the association of daily pain and religious coping. Within individuals, pain was significantly lower on days when religious coping was not used. Implications: Results contribute to a growing understanding of how individuals use their religious beliefs to cope with daily pain and perhaps, to the formulation of more targeted therapies for individuals living with chronic illness. (Supported by R01-AG041655 D. Smith and P. Parmelee, PIs.)

